# Reduced recruitment of 53BP1 during interstrand crosslink repair is associated with genetically inherited attenuation of mitomycin C sensitivity in a family with Fanconi anemia

**DOI:** 10.18632/oncotarget.23375

**Published:** 2017-12-17

**Authors:** Emilie Lesport, Alina Ferster, Armand Biver, Benoit Roch, Nadia Vasquez, Nada Jabado, Francina Langa Vives, Patrick Revy, Jean Soulier, Jean-Pierre de Villartay

**Affiliations:** ^1^ Laboratory “Genome Dynamics in The Immune System”, INSERM UMR1163, Université Paris Descartes Sorbonne Paris Cité, Institut Imagine, Paris, France; ^2^ Departement d’Hémato-Oncologie, Hôpital Universitaire des Enfants Reine Fabiola, Bruxelles, Belgium; ^3^ Service de Pédiatrie Générale, Centre Hospitalier De Luxembourg, Luxembourg; ^4^ INSERM U944, Institut Universitaire d’Hématologie, Paris, France; ^5^ Department of Human Genetics and Department of Experimental Medicine, McGill University, Montreal, Canada; ^6^ Centre d’Ingénierie Génétique Murine, Institut Pasteur, Paris, France

**Keywords:** Fanconi anemia, DNA interstrand crosslinks, DNA double strand break repair, 53BP1, ATM

## Abstract

The Fanconi anemia (FA) pathway is implicated in the repair of DNA interstrand crosslinks (ICL). In this process, it has been shown that FA factors regulate the choice for DNA double strand break repair towards homologous recombination (HR). As this mechanism is impaired in FA deficient cells exposed to crosslinking agents, an inappropriate usage of non-homologous end joining (NHEJ) leads to the accumulation of toxic chromosomal abnormalities. We studied a family with two FANCG patients and found a genetically inherited attenuation of mitomycin C sensitivity resulting *in-vitro* in an attenuated phenotype for one patient or in increased resistance for two healthy relatives. A heterozygous mutation in ATM was identified in these 3 subjects but was not directly linked to the observed phenotype. However, the attenuation of ICL sensitivity was associated with a reduced recruitment of 53BP1 during the course of ICL repair, and increased HR levels. These results further demonstrate the importance of favoring HR over NHEJ for the survival of cells challenged with ICLs.

## INTRODUCTION

Fanconi anemia (FA) is a rare genetic disorder characterized by early bone marrow failure (BMF), congenital abnormalities, and predisposition to malignancies. To date, 21 genes have been implicated in the diagnosis of FA. These genes encode proteins known to participate to the repair of DNA interstrand crosslinks (ICL) [1, 2]. The proteins of the FA pathway can be divided into three functional groups: the FA core complex, the FANCD2/FANCI complex and the downstream effectors. FA core complex proteins FANCA, FANCB, FANCC, FANCE, FANCF, FANCG together with the E2 ubiquitin conjugating enzyme FANCT/UBE2T and the E3 ubiquitin ligase FANCL are necessary for the monoubiquitination of the FANCD2/FANCI complex. This critical step in the repair of ICL allows the recruitment of the downstream effectors including FA proteins FANCD1/BRCA2, FANCJ/BRIP1, FANCN/PALB2, FANCO/RAD51C, FANCP/SLX4, FANCQ/XPF, FANCR/RAD51, FANCS/BRCA1, FANCU/XRCC2 and FANCV/REV7, resulting in the resolution of the crosslink by homologous recombination (HR). Inactivation of the FA pathway induces HR defects and inappropriate usage of the non-homologous end joining (NHEJ) for the repair of DNA double-strand breaks (DSBs) created at crosslinks, leading to translocations and other genome rearrangements [3]. Accordingly, accumulation of chromosomal abnormalities and acute sensitivity to ICL-inducing drugs such as mitomycin C (MMC) or cisplatin are two hallmarks of FA cells.

Recently, it has become clear that the FA pathway plays an important role to favor HR over NHEJ during the repair of ICLs [4–6]. NHEJ and HR are the two main pathways utilized by cells to achieve DSB repair. To ensure genomic stability, the balance between these two pathways is tightly regulated and cell-cycle dependent [7, 8]. During the G1 phase, HR is not amenable and NHEJ directly ligate the two ends of the break in an error-prone fashion. In this context, the DNA damage response (DDR) factor 53BP1 and its effector RIF1 accumulate at DSBs were they inhibit extensive resection of DNA ends and initiation of HR [9–11]. In contrast, DSBs formed during S or G2 phases are preferentially repaired by HR, using the sister chromatid as repair template. Here, BRCA1 and CtIP are two important factors to prevent RIF1 accumulation and initiate resection to create HR-prone substrates [12, 13]. In the specific context of ICL repair, it has been shown that an active FA pathway favors HR by preventing 53BP1 accumulation at damaged chromatin [6] and promoting CtIP recruitment via FANCD2 [14, 15]. Some evidences also indicate that depletion or inhibition of NHEJ factors in FA-deficient cells could suppress, at least partially, their ICL sensitivity, further demonstrating the deleterious effects of inappropriate usage of NHEJ during ICL repair [5, 4].

Here, we describe a natural condition of *in-vitro* attenuation of ICL sensitivity in FANCG mutated cells from a FA patient, found to be an autosomal dominant genetic trait inherited from his healthy mother. Although we identified a heterozygous mutation in the *ATM* gene for the attenuated patient and his mother and healthy sister, we could not formally link this mutation to the ICL-resistant phenotype. Moreover, we show that the identified mutation does not impair the function of ATM in the DNA damage response *in-vitro* nor *in-vivo*. However, we demonstrate that the attenuation of ICL sensitivity is likely due to a modified balance between NHEJ and HR for DSB repair, independent of the FA pathway.

## RESULTS

### Attenuation of MMC sensitivity observed in a family with Fanconi anemia

**Figure 1:**
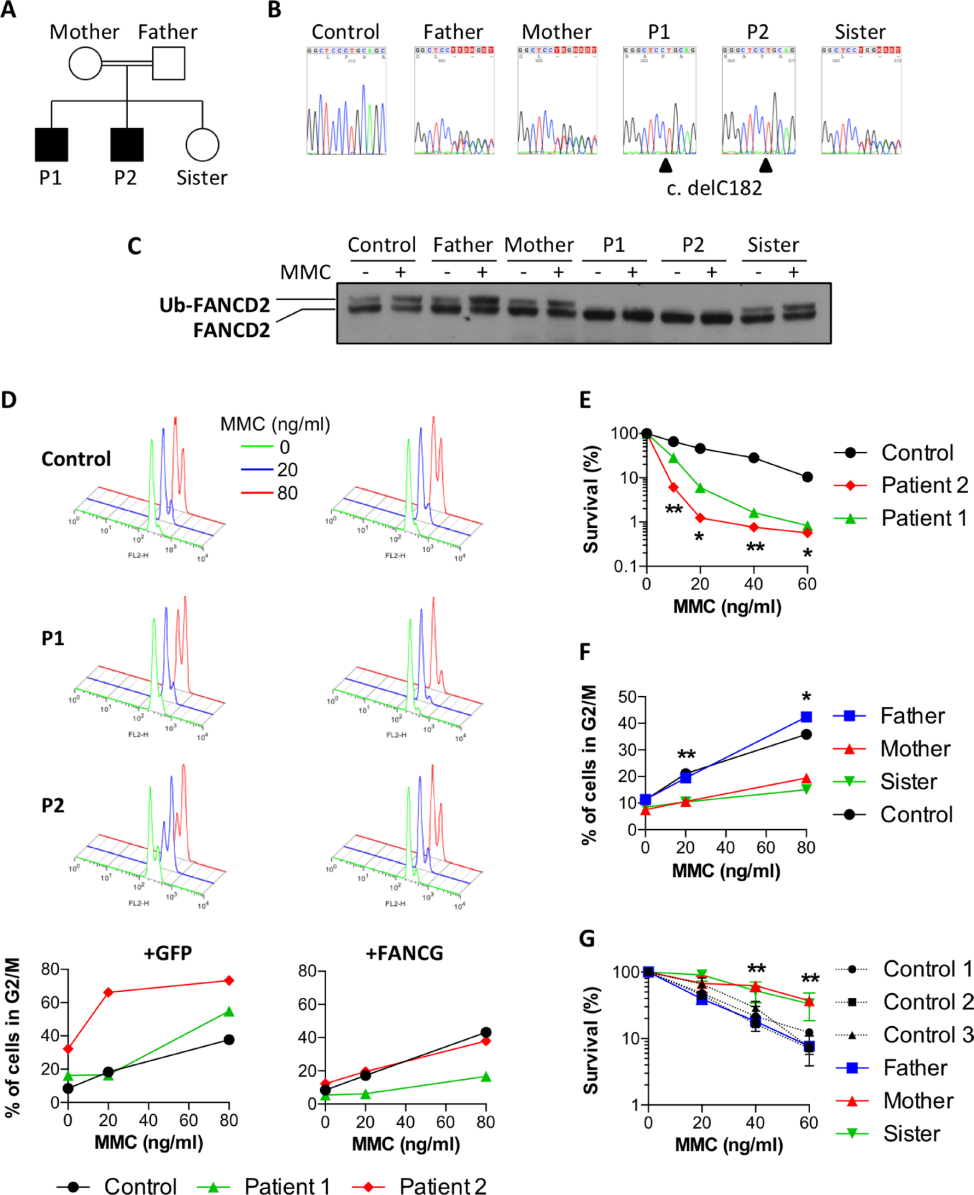
Attenuation of MMC sensitivity in a family with Fanconi anemia (**A**) Pedigree of the family. Black boxes indicate the two FA patients. (**B**) Sanger sequencing showing delC182 in FANCG found to be homozygous in P1 and P2, and heterozygous in the parents and the healthy sister. (**C**) FANCD2 immunoblot performed with fibroblasts exposed or not to 150 ng/ml MMC for 18 h. (**D**) Primary fibroblasts from a healthy control and from patients P1 and P2 were transduced either with GFP (left panels) or with wild-type FANCG (right panels) containing vectors. The upper histograms show cell cycle analysis of cells treated for 48h with 0, 20 or 80 ng/ml MMC (green, blue and red histograms respectively). The percentages of cells in G_2_ phase are plotted on the bottom panels. (**E**) Cell survival of P1, P2 and a healthy control in presence of increasing doses of MMC. Results shown as mean ± SD of triplicates are representative of 3 independent experiments. Statistical analyses were performed using unpaired *t*-tests.(**F**) Percentages of primary fibroblasts from the father, mother, sister, and a healthy control accumulating in G_2_ phase 48 h after treatment with the indicated doses of MMC. Results are representative of 3 independent experiments. Statistical analyses were performed using unpaired *t*-tests comparing the mean of the mother and sister versus the mean of the father and control. (**G**) Cell survival of the father, mother, sister and 3 healthy controls in presence of increasing doses of MMC. Results shown as mean ± SD of triplicates are representative of 3 independent experiments. Statistical analyses were performed using unpaired *t*-tests comparing the mean of the mother and sister versus the mean of the 3 controls. ^*^P < 0.05, ^**^P < 0.01, ^***^P < 0.001.

The two sons from the studied family, patients P1 and P2 (Figure 1A), born from consanguineous parents of Nepalese origin, were initially investigated for suspicion of a BMF syndrome (Table 1). Aged 8 and 5 at the time of diagnosis, they presented with an extremely short stature, microcephaly, cytopenia, and a low bone marrow cellularity. Due to further degradation of their cytopenia, the two patients subsequently underwent hematopoietic stem cell transplantation (HSCT). This clinical presentation was highly evocative of Fanconi anemia syndrome and a homozygous mutation in the FANCG gene was indeed identified in both P1 and P2 upon whole exome sequencing (WES). The mutation consists in the deletion of one nucleotide (NM_004629.1:c.182del) (Figure 1B), which creates a frameshift leading to the appearance of a premature stop codon (p.Pro61Leufs*11), thus abrogating the expression of a functional FANCG protein. To the best of our knowledge, this particular mutation in FANCG was not previously described (http://databases.lovd.nl/shared/variants/FANCG), although a one nucleotide deletion 3 nucleotides upstream (c.179delT), which results in essentially the same loss of function allele was reported in a South African FA patient [16]. The expected consequence of this FANCG loss of function is the absence of FANCD2 monoubiquitination in P1 and P2 cells upon MMC treatment or not (Figure 1C). In contrast, the healthy father, mother, and sister, all carrying the heterozygous c.182del mutation, presented a normal monoubiquitination of FANCD2 in protein extracts from their fibroblasts (Figure 1B, 1C).

**Table 1:** Patients’ clinical features at diagnosis

	Patient 1	Patient 2	Sister
Age	8 y 9 mo	5 y 3 mo	2 y
Sex	Male	Male	Female
Weight (kg)	19.7 (–2.7)	11.8 (–4.7)	11 (–1.0)
Head circumference (cm)	49 (–3.2)	44 (–5.9)	NA
Height (cm)	117 (–2.6)	94 (–3.8)	86 (–0.1)
Body mass index	14.4 (–1.1)	13.4 (–2.2)	14.9 (–1.5)
Hemoglobin (g/L)	94	112	122
MCV (fL)	103	116	79
Platelets (×10^9^/L)	45	65	382
WBC (×10^9^/L)	2.97	3.96	7.93
PMN (×10^9^/L)	0.9	1.31	2.43
Lymphocytes (×10^9^/L)	1.66	2.56	4.85
Fetal hemoglobin (%)	13	16	0.7
α-Fetoprotein	<15	<15	NA
Marrow examination	Low cellularity Dyserythropoiesis Erythrophagocytosis	Low cellularity Dyserythropoiesis	Not performed
Cytogenetics	46 XY, –7[1]/46, XY[20]	46 XY, chtb (3), p21[4]/46XY[16]	Not performed

*MCV*, mean corcuspucal volume; *WBC*, white blood cell; *PMN*, polymorphonuclear.

Values in parentheses indicate SD.

Although the two patients shared the same FANCG mutation and both presented clinical features characteristic of FA, *in-vitro* assays revealed a striking difference in MMC sensitivity of their respective fibroblasts. The primary fibroblasts derived from P1 and P2 were first assayed for the MMC-induced G_2_/M cell cycle arrest, a characteristic abnormality of FA cells. As shown in Figure 1D, whereas P2 cells demonstrated an important block at G_2_/M already at the initial dose of 20ng/ml MMC, cells from P1 were undistinguishable from controls at this dose, demonstrating an increased cell cycle arrest at the following dose of 80 ng/ml. Likewise, the MMC sensitivity of P1 SV40 transformed fibroblasts was significantly milder than that of P2 (Figure 1E). The transduction of P2 cells with a lentivirus vector restoring the expression of wild-type FANCG, but not with a GFP expression vector alone, abrogated the increased MMC-induced G_2_ phase arrest to a level comparable to that of control cells, validating the FANCG c.delC182 mutation as the cause of the MMC sensitivity in these cells (Figure 1D). Interestingly, the residual MMC-induced G_2_/M cell cycle arrest in FANCG-complemented fibroblasts from P1 was lower when compared to his brother P2 and the control. This observation was repeated on a second, independently derived, series of fibroblast lines, ruling out a cell culture bias (data not shown). In addition to these differences observed *in-vitro*, it is noticeable that the growth retardation and microcephaly of P2 were somehow more severe (Table 1) and that the BMF appeared earlier in age than his brother (8y instead of 11y).

Altogether this first series of observations suggested either a phenotypic attenuation of the FANCG defect in P1 resulting in a milder MMC sensitivity *in vitro* and a least clinical impact, or, on the contrary, an exacerbated FA phenotype in P2. To appreciate whether a genetic/inheritable trait was responsible for this phenotypic difference we analyzed the various family members for their MMC sensitivity (Figure 1F and 1G). The fibroblasts from the father exposed to MMC presented a moderate G_2_ arrest and a cell survival comparable to several control lines. Surprisingly however, the MMC-induced G_2_ phase arrest was almost abrogated in the fibroblasts from the mother and sister, and comparable to the P1 complemented fibroblasts. Likewise, the survival of mother and sister fibroblasts in presence of MMC was significantly higher than that of many control cells (Figure 1F and 1G).

Altogether, these results indicated that the mother transmitted a dominant “ICL-resistance” character to her daughter and to P1. This genetic trait would partially compensate the absence of a functional FA pathway in P1 cells, whereas it would further increase the resistance to MMC in FA-proficient cells.

### The attenuation of sensitivity to MMC is not due to a major NHEJ defect

**Figure 2:**
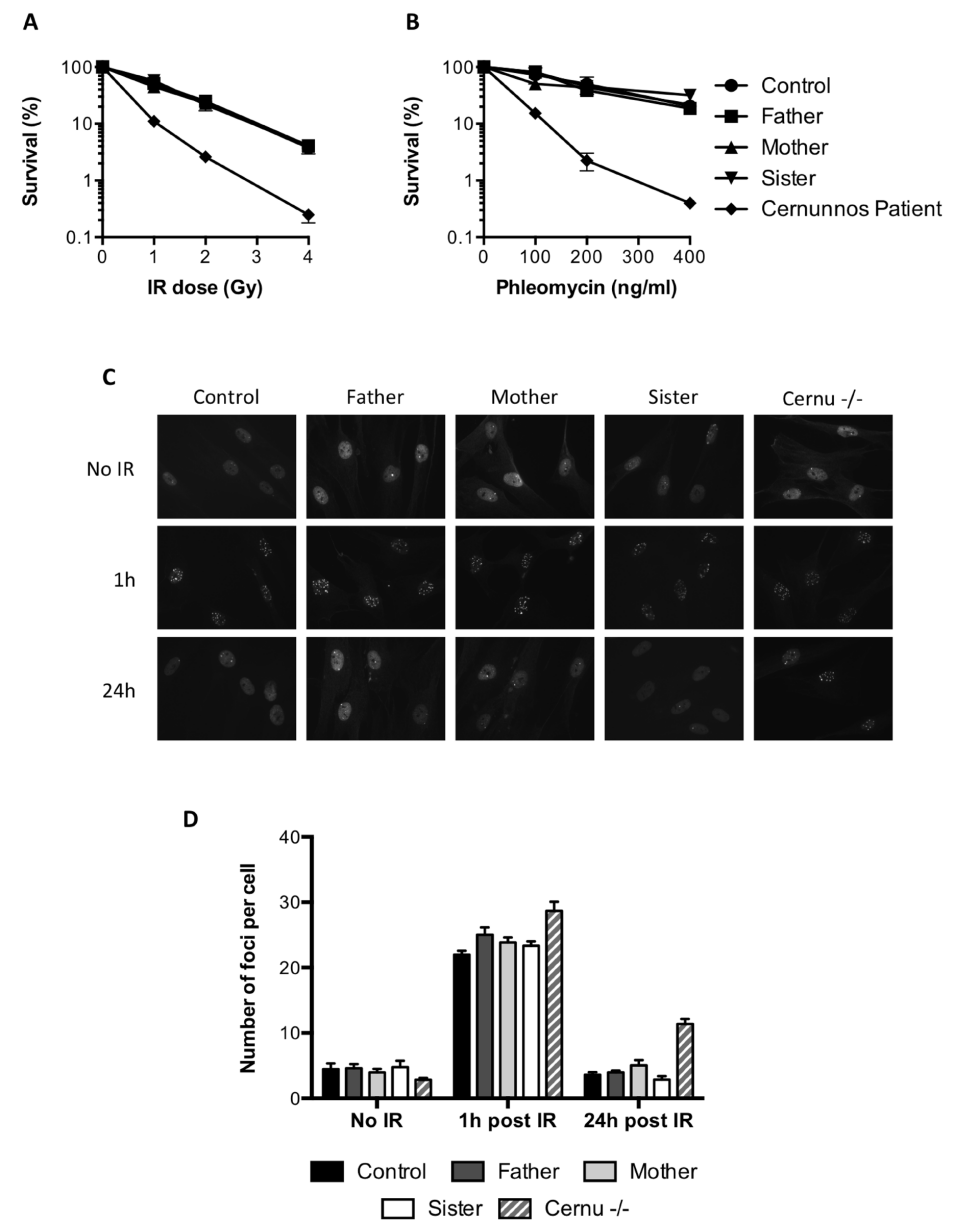
Functional NHEJ in the MMC-resistant individuals **(A**, **B**) Survival of the father, mother, sister, a healthy control and a Cernunnos deficient patient exposed to increasing doses of ionizing radiations (A) or phleomycin (B). Results shown as mean ± SD of duplicates are representative of three independent experiments. **(C**, **D**) Primary fibroblasts from the father, mother, sister, a healthy control and a Cernunnos deficient patient (Cernu–/–) were either left untreated or irradiated with 0.5 Gy and further incubated for 1 or 24 h before fixation and 53BP1 immunostaining. (**C**) Representative images of 53BP1 staining. (**D**) Number of 53BP1 foci per cell (mean ± SEM) quantified in at least 50 nuclei from 3 independent experiments.

It has been shown that the ICL sensitivity of FA cells could be rescued by inhibiting NHEJ [4, 5]. The lack of a critical NHEJ factor in the mother or sister cells was very unlikely in the absence of any clinical phenotype. To further exclude this hypothesis, we investigated the response of the cells to ionizing radiations (IR)-induced DSBs. As shown in Figure 2A, the survival of the mother and sister cells was similar to that of the father or control cells upon irradiation, whereas fibroblasts from a NHEJ-deficient Cernunnos patient showed an acute sensitivity to IR. The same observations were made using the radio-mimetic drug phleomycin to induce DSBs (Figure 2B). We also studied the kinetics of 53BP1 foci formation and resolution following IR. As shown in Figure 2C, all cells tested exhibited 53BP1 IR-induced foci 1h after irradiation. These foci almost completely disappeared at 24 hours, except for the Cernunnos patient (Figure 2C and 2D).

In regard of all these results, we concluded that the NHEJ pathway was functional in the MMC-resistant individuals.

### The attenuation of MMC sensitivity is associated with a modified balance between NHEJ and HR

**Figure 3:**
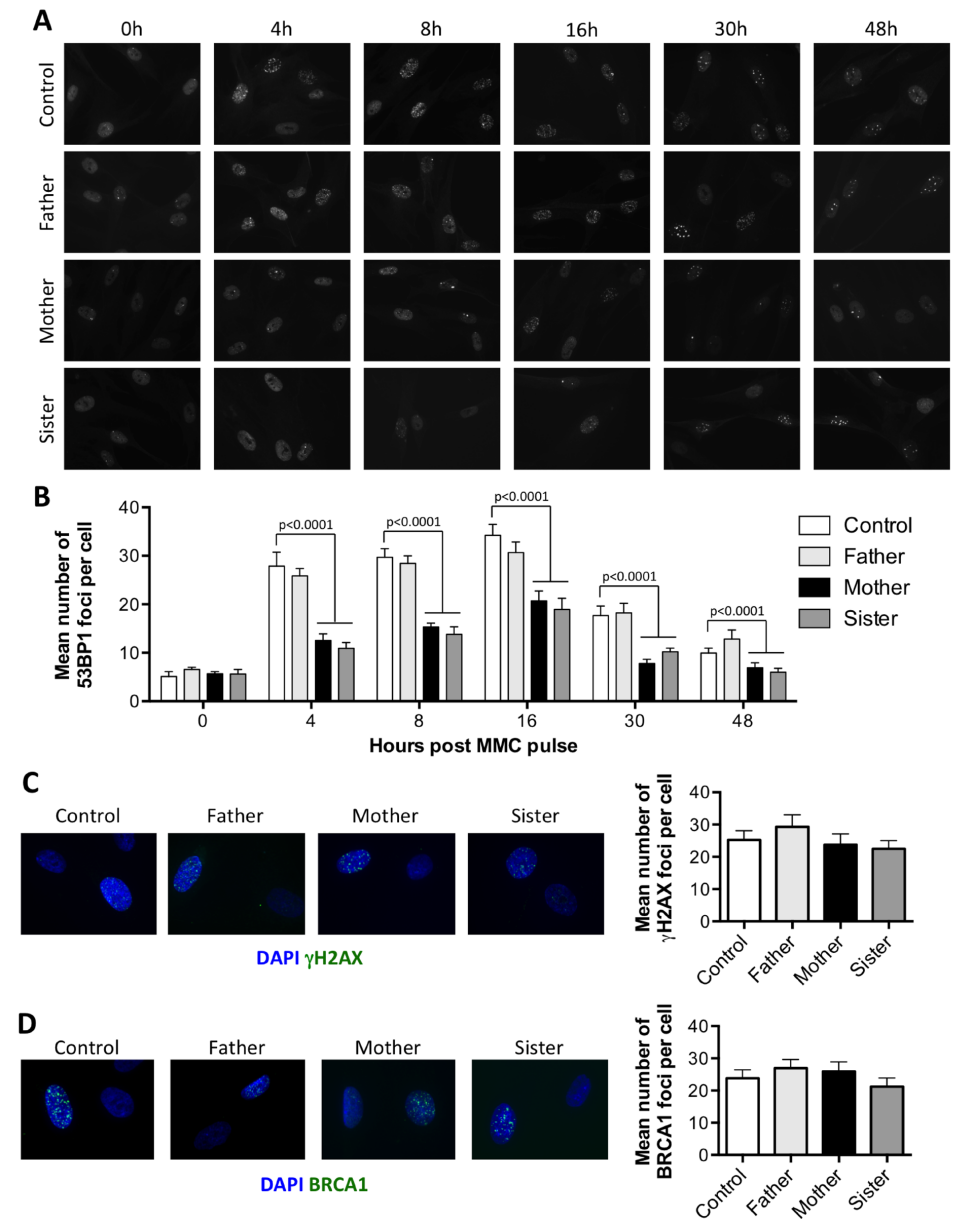
The attenuated sensitivity to MMC is associated with a reduced recruitment of 53BP1 at ICLs (**A–B**) Recruitment of 53BP1 following MMC treatment. Primary fibroblasts from the father, mother, sister, and a healthy control were pulsed for 1 hour with 1 μg/ml MMC and further incubated for the indicated times before fixation and 53BP1 immunostaining. (A) Representative images. (B) Number of foci per cell as quantified from at least 100 nuclei from 3 independent experiments, shown as mean ± SEM. Statistical analyses were performed using Mann-Whitney tests comparing each sample with the control at the corresponding time point. (**C**–**D**) Cells treated as in A were incubated for 16 h before fixation and γH2AX (C) or BRCA1 (D) immunostaining. Left panel show representative images. Right panel show the mean number of foci per cell calculated as in B.

It has been proposed that FA proteins prevent the recruitment of NHEJ factors during the repair of ICL to favor HR. As 53BP1 plays a key role in the choice for DSB repair pathway [17], we analyzed the recruitment of this factor during the repair of ICL. Primary fibroblasts, pulsed for 1 hour with 1 µg/ml MMC, were stained for 53BP1 at different time points (Figure 3A). Around 30 53BP1 foci per cell were observed as soon as 4 h following the MMC pulse in control cells. The amount of 53BP1 foci then slightly increased to reach a peak at 16 h, before starting to decrease as the ICLs are repaired. In P1 and P2 cells however, the 53BP1 foci accumulated over time, as expected for FA deficient cells [6] (Supplementary Figure 1). The kinetics and intensity of 53BP1 foci formation in the father’s cells were identical to that observed in control cells. In contrast, although the kinetics of 53BP1 foci formation was similar in mother’s and sister’s cells, with a maximum reached at 16 h, the mean number of foci per cell was always significantly lower at all time points tested (Figure 3B).

The lower levels of 53BP1 foci observed in MMC treated P1 cells compared to P2 cells could be due to a longer division rate (data not shown) resulting in less ICL-induced DSBs. In the mother and sister’s cells however, the reduced recruitment of 53BP1 foci could not be explained by a reduced amount of DSBs, as γH2AX foci were present in similar amount in all the cells (Figure 3C).

As BRCA1 is known to antagonize 53BP1 recruitment at DSBs, thus favoring HR pathway [7], we analyzed the recruitment of BRCA1 following MMC treatment. As shown in Figure 3D, BRCA1 foci were as abundant in the mother or sister’s cells as in the control and father’s cells 16 h following the MMC pulse. Thus, the reduced recruitment of 53BP1 is not caused by an increased recruitment of BRCA1.

**Figure 4:**
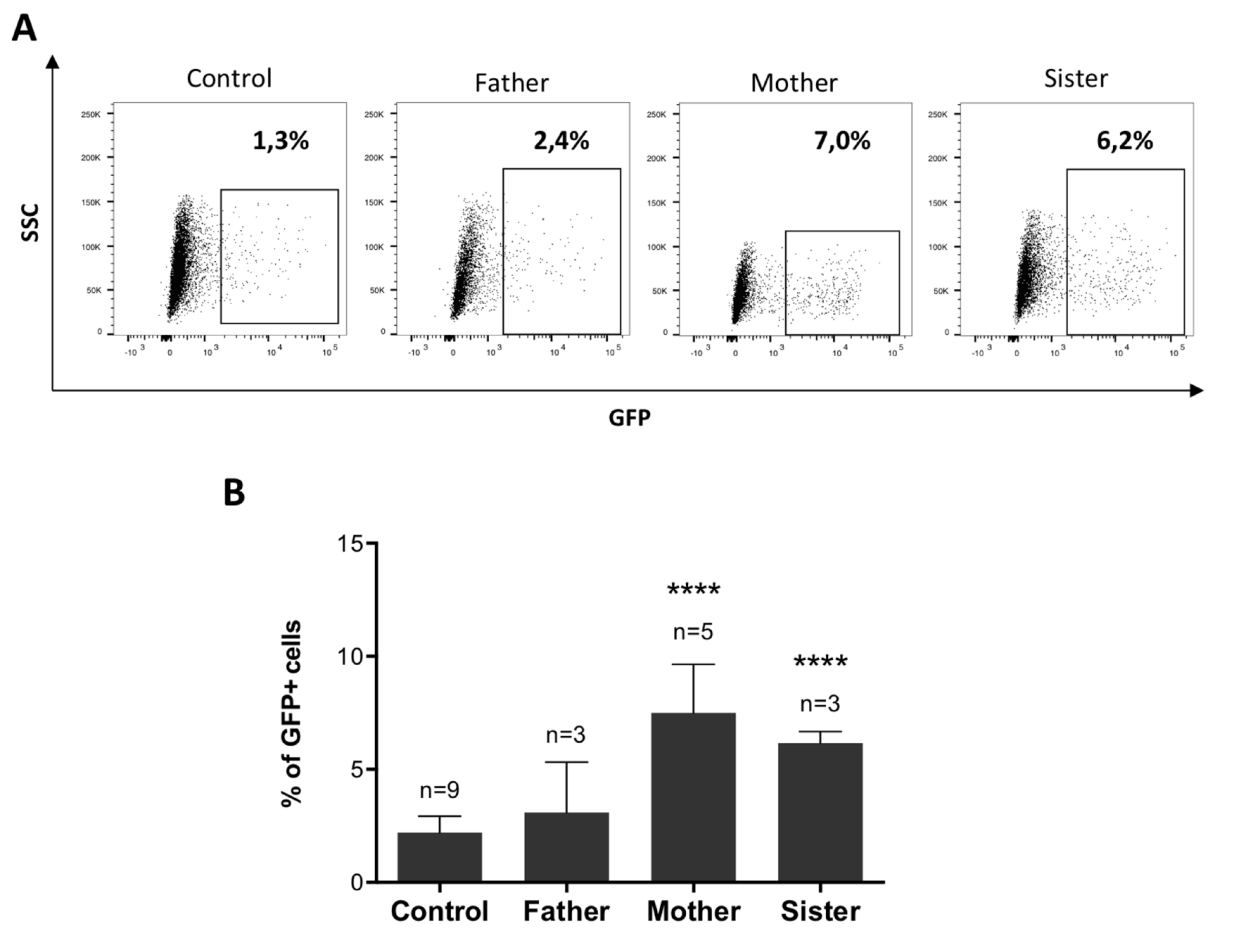
The attenuated sensitivity to MMC is associated with enhanced HR (**A–B**) HR levels measured with the DR-GFP reporter. DR-GFP cell lines were co-transfected with RFP and I-SceI expression plasmids (ratio 10:1) and the percentage of GFP^+^ cells was analyzed by FACS. (A) Representative dotplots showing GFP expression within the RFP^+^ population. (B) Percentages of GFP^+^ cells calculated among RFP^+^ cells. Data shown are mean ± SD from at least 3 replicates from independent experiments. Statistical analyses were performed using unpaired *t*-tests. ^****^P < 0.0001.

With a lesser engagement of 53BP1, we assumed that the HR pathway should be favored over NHEJ for the repair of DSB. To test this hypothesis, we generated cell lines with a stably integrated DR-GFP substrate from the fibroblasts of the father, mother, sister, and control cells. As shown in Figure 4, following induction of a DSB in the DR-GFP cassette by I-SceI cut, the percentages of GFP^+^ cells were significantly increased in the mother and sister cells as compared to the control, attesting for an augmented HR in these cells.

From these experiments we conclude that the observed attenuation of ICL sensitivity in mother’s and sister’s cells may result from a shift towards HR during DSB repair pathway choice, associated with a reduced recruitment of 53BP1 at ICL sites.

### The attenuation of MMC sensitivity is not due to a heterozygous mutation in the ATM gene

In order to identify a genomic variant responsible for the attenuated FANCG phenotype observed in this family, we performed whole exome sequencing, in search of an autosomal dominant heterozygous mutation shared by P1, his mother, and his sister, but absent in P2 and his father. Among 92 candidate variants predicted by this analysis, we decided to focus on a missense mutation (c.94C>T, p.R32C) in the exon 3 of the *ATM* gene. ATM is a protein kinase known to play a key role in the DDR and has been implicated in the regulation between NHEJ and HR for DSB repair. Moreover, the predicted mutation stands just downstream of the TAN motif, previously described to be important for the response to DNA damage in Tel1, the S. cerevisiae ortholog of ATM [18].

**Figure 5:**
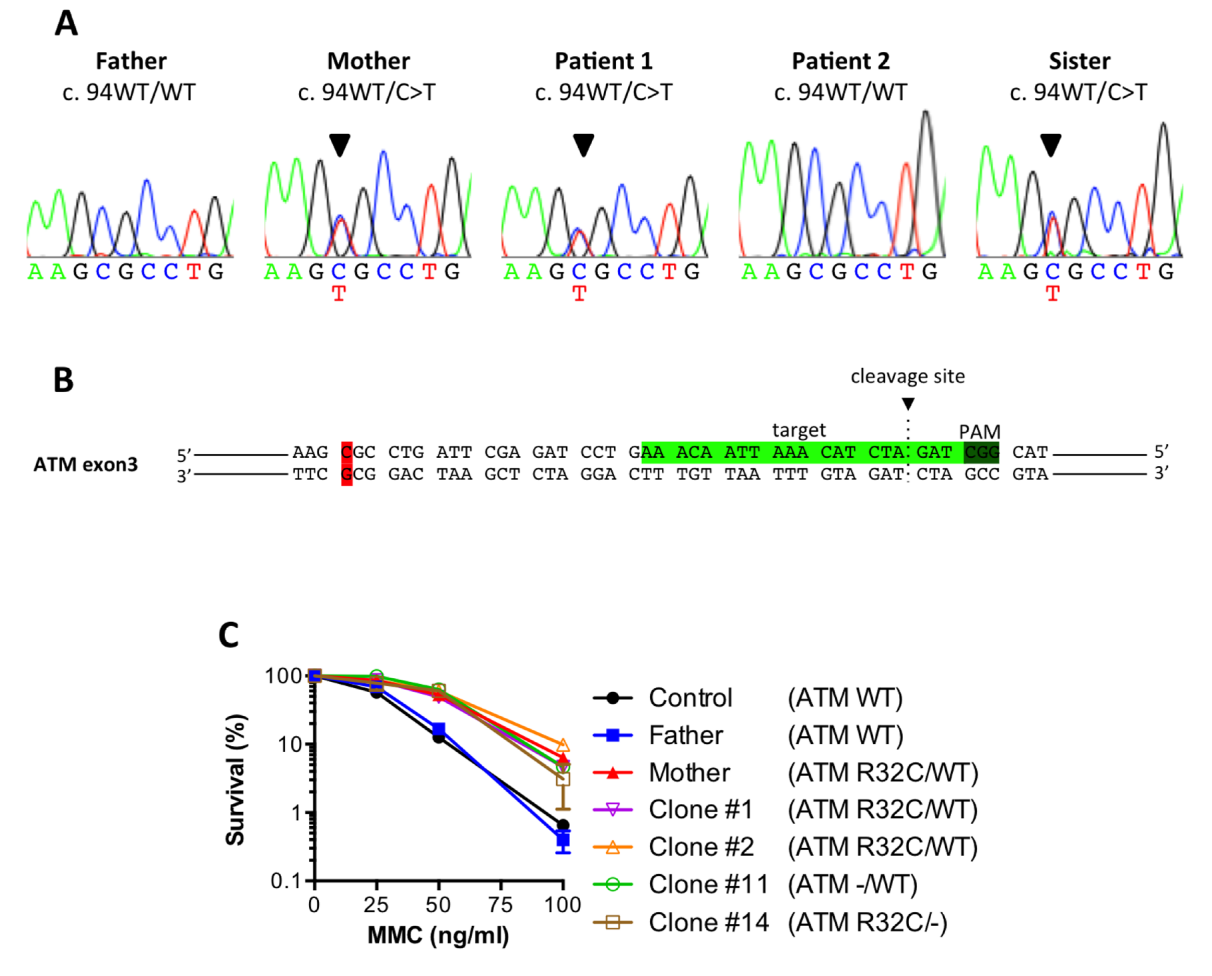
The ATM R32C mutant allele found in MMC-resistant individuals is dispensable for the enhanced survival (**A**) Sanger sequencing showing a heterozygous 94C>T mutation in the *ATM* gene for the mother, P1 and its sister. (**B**) CRISPR-Cas9 target sequence in exon 3 of human ATM. (**C**) MMC survival of a healthy control, the father, the mother and clones #1, 2,11 and 14. Results shown as mean ± SD of duplicates are representative of 3 independent experiments.

Sanger sequencing confirmed that the mother carried the heterozygous c.94C>T mutation and that it had been transmitted to P1 and his sister (Figure 5A). This mutation resulted in the substitution from arginine 32 to cysteine, and was predicted to be probably damaging by Polyphen (score 0,998) and CADD (Phred score of 26). It is a rare variant (dbSNP: rs148061139) with an overall frequency of 0.012% and 0.06/0.02% in South Asian and East Asian populations respectively (http://exac.broadinstitute.org/variant/11-108098524-C-T).

To formally link this ATM variant to the attenuated phenotype, we decided to disrupt the R32C allele in the mother cells by means of CRISPR/Cas9 genome engineering. The SV40 fibroblasts from the mother were transfected with the pX330 plasmid allowing the expression of the Cas9 together with a gRNA targeting the exon 3 of *ATM* (Figure 5B) chosen to be close to the 94C>T variant to facilitate screening of the disrupted alleles. Several clones were derived from the bulk of transfected cells and screened by sequencing. Part of the clones showed no mutagenesis and were still heterozygous (ATM R32C/WT), whereas others had lost either their R32C allele (ATM –/WT) or their WT allele (ATM –/R32C) (Table 2). Sanger sequencing of the genomic DNA and cDNA established that the clone #11 had lost its R32C allele due to a 37bp deletion, causing a frameshift and a premature stop codon in the *ATM* gene. The same analyses performed on clone #14 revealed the c.94C>T variant as homozygous, suggesting a large deletion on the wt allele, precluding its PCR amplification with the used oligonucleotides. We concluded that the clone #14 had lost the WT allele and only expresses an ATM protein bearing the R32C mutation. We performed a MMC survival assay using clones #1, 2,11 and 14. As shown in Figure 5C, the heterozygous clones (#1 and 2) and the clones that had lost either the R32C allele (#11) or the WT allele (#14) were all highly resistant to MMC, just like the untargeted mother cells. Thus, the loss of the R32C variant of ATM could not restore a regular sensitivity to MMC to the cells. This result argued that the R32C mutation of ATM was dispensable to induce ICL resistance.

**Table 2:** Characteristics of clones obtained by CRISPR/Cas9 mediated ATM inactivation in the mother’s fibroblasts

Clone	Cas9 induced mutagenesis		Genotype
	On WT allele	On R32C allele	
#1	None	None	R32C/WT
#2	None	None	R32C/WT
#11	37bp deletion	None	–/WT
#14	None	Allele not amplified	R32C/–

### The R32C ATM variant has a functional DDR activity

**Figure 6:**
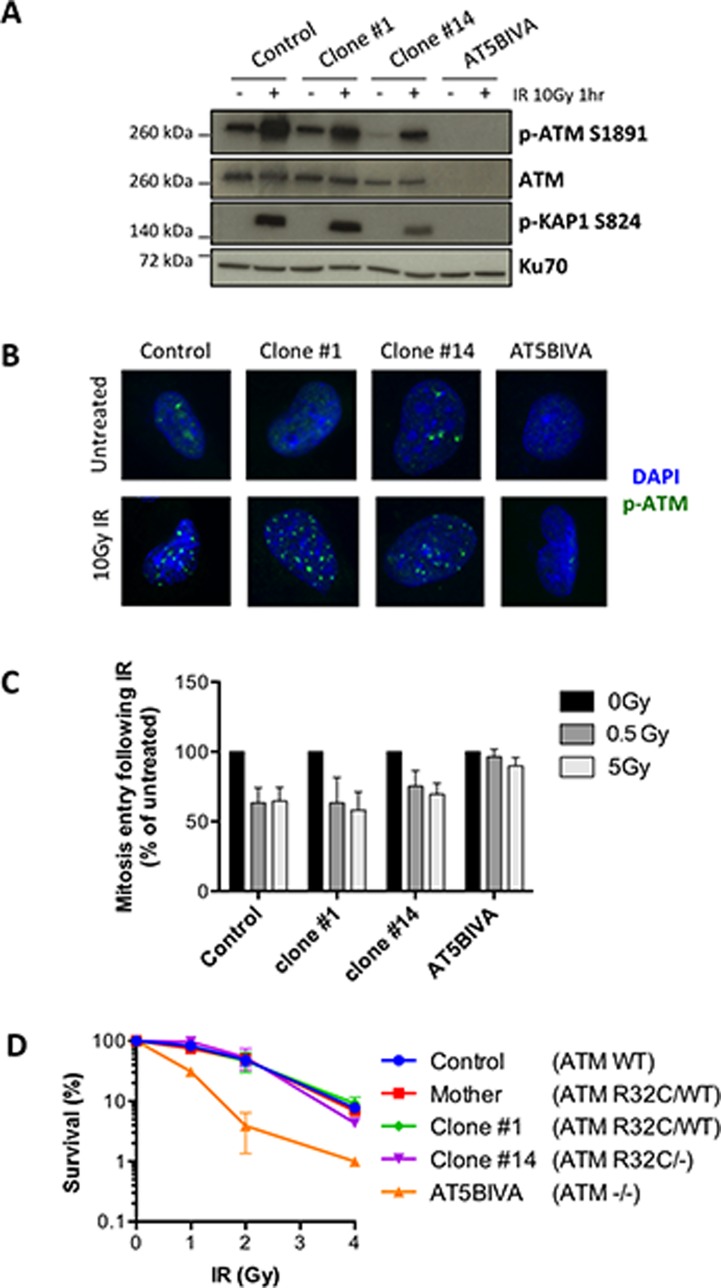
The R32C variant of ATM is functional for the DNA damage response (**A–D**) DDR analyses in SV40-transformed fibroblasts from a healthy control, the mother, the ATM R32C/WT clone #1, the ATM R32C/- clone #14, and in AT5BIVA cell line. (A) Western blot analysis of the IR-induced phosphorylation of ATM at S1981 and Kap1 at S824 one hour after 10 Gy irradiation. (B) Representative images showing phospho-ATM S1981 IR-induced foci (green staining) one hour after 10 Gy irradiation. DAPI: blue staining. (C) Percentages of G_2_ cells entering mitosis following 0.5 or 5 Gy irradiation calculated related to untreated. Results are presented as mean ± SD of 3 independent experiments. (D) Cell survival after exposure to increasing doses of IR. Results shown as mean ± SD of duplicates are representative of 3 independent experiments.

In order to determine the putative consequences of the R32C mutation for the canonical ATM function in DDR, we took advantage of the clone #14 that expresses only the R32C variant. We first analyzed the auto-phosphorylation of the protein at serine 1981 by WB and IF upon DNA damage. ATM immunoblot (Figure 6A) showed reduced levels of ATM, consistent with the loss of one allele. The IR-induced S1981 phosphorylation of ATM was only reduced by half in the monoallelic clone #14 compared to control cells and AT5BIVA fibroblasts used as fully ATM defective control, arguing for a functional kinase activity of the R32C variant. This was further confirmed by the efficient ATM-dependent phosphorylation of Kap1, one key ATM substrate, following IR. Moreover, p-ATM IRIFs were detected for both clones #1 and 14 following 10Gy IR (Figure 6B). To further investigate the functionality of ATM R32C, we analyzed the G2/M cell cycle checkpoint upon IR. The phosphorylation of histone H3, indicative of mitosis entry, was reduced in irradiated control cells as expected, as well as in all the clones tested, whereas it was unaffected in the cell cycle checkpoint defective AT5BIVA cells (Figure 6C). Thus, ATM R32C is as proficient as WT ATM to induce a checkpoint arrest upon DNA damage. Lastly, we analyzed the survival of the different clones to IR. Unlike AT5BIVA cells, which demonstrate an increased cellular sensitivity to IR, the clone #14 has no acute sensitivity to IR, with a survival curve similar to that of control cells (Figure 6D).

We also generated ATM R32C homozygous mice using CRISPR/Cas9 genome engineering (Supplementary Figure 2A). These mice were viable, presented no congenital abnormalities, and were also fertile, both males and females. Immunophenotyping of the spleen and thymus revealed no developmental defects in R32C/R32C mice (Supplementary Figure 2B and 2C). Moreover, the response to IR of R32C homozygous T cells was normal in regard of Kap1 phosphorylation, unlike ATM –/– T cells (Supplementary Figure 3).

Altogether, these results indicate that the R32C mutation in ATM does not abrogates its function in DDR.

## DISCUSSION

In the present study, we report a case of inherited attenuation of MMC sensitivity in a family with FA. First highlighted by the different outcome observed with the cells from two brothers sharing the same FANCG homozygous mutation, an MMC resistant phenotype was also identified in the healthy mother’s and sister’s cells. We took advantage of these resistant cells to try to decipher the underlying mechanism in a simple context, without interference caused by the FANCG mutated background. Although the genetic origin of the MMC resistance remains to be identified, we showed that it is associated with a reduced recruitment of 53BP1 in the context of ICL repair and elevated HR levels. Altogether, these results point toward a shift in the balance between NHEJ and HR for the repair of DSBs.

Our first observations on patient P1 revealed that even with a lack of FANCD2 monoubiquitination as expected for FA core complex deficient cells, the MMC induced G2/M cell cycle arrest and the MMC toxicity were significantly reduced compared to his brother presenting the same FANCG homozygous mutation (Figure 1A–1E). A subset of FA patients presenting a defective FANCD2 monoubiquitination but an abrogation of G2/M checkpoint has been previously described [19]. This attenuation phenotype was linked to the clonal selection of PBLs with a downregulated ATR-Chk1 checkpoint pathway, allowing cell survival despite unrepaired DNA damage. Unlike the attenuated patients described by Ceccaldi and colleagues, patient P1 phenotype was not restricted to hematopoietic cells and was rather a constitutive, genetically inherited trait. In line with this, we observed an increased resistance to MMC for healthy mother and sister of P1 (Figure 1F and 1G). These data suggested a shared mechanism that would synergize with a functional ICL repair pathway in the mother and sister cells to further increase resistance to MMC, while partially compensating the deficient FA pathway in P1 cells.

Recent works suggest that inhibition of NHEJ can partially rescue the MMC-induced toxicity in FA cells by preventing chromosomal aberrations [4, 5]. In the cells studied, a normal response to IR-induced DNA damage suggested a functional NHEJ pathway (Figure 2). However, we found that the attenuation phenotype was linked to a reduced recruitment of 53BP1 in the course of ICL repair. The formation of 53BP1 foci upon MMC treatment followed the same kinetics in all the cells tested, but in average, the number of foci was lower in resistant cells compared to normal cells (Figure 3A and 3B). In patients’ cells, we observed an accumulation of 53BP1 foci over time consistent with the FANCG deficiency [6], although in reduced levels in P1 compared to P2 cells (Supplementary Figure 1). Since the initiation of ICL repair mainly occurs during S phase, a decreased number of 53BP1 foci could be explained by a lower occurrence of DSBs in slowly dividing cells. We indeed noticed a longer doubling time in P1 primary fibroblasts that could at least partially explain the lower amounts of 53BP1 foci observed. In contrast, the division rates were not decreased for the resistant cells (data not shown). Moreover, the number of γH2AX and BRCA1 foci upon MMC treatment were similar in all the cells tested, indicating the same amount of DSBs (Figure 3C and 3D).

53BP1 binds chromatin at dimethylated histone H4K20 via its tandem Tudor domains [17]. Recently, it has been shown that activated FANCD2 promotes H4K16 acetylation by TIP60 to block the recruitment of 53BP1 at H4K20me2 [6]. This mechanism is most probably active in the FA-proficient mother and sister cells, although the extra decrease in the number of 53BP1 foci suggests an additional mechanism that antagonizes 53BP1 recruitment.

53BP1 localized at DSBs recruits RIF1 to promote repair by NHEJ [20]. With a decreased number of 53BP1 foci, we hypothesize that the amount of RIF1 foci would be reduced likewise. Unfortunately, we failed to detect the formation RIF1 foci following MMC treatment (data not shown). This could be explained by the very transient recruitment of RIF1 during the G2 phase of the cell cycle [21], when the repair of MMC-induced damage likely occurs.

The elevated levels of HR measured in the resistant cells (Figure 4) suggest that the reduced recruitment of 53BP1 contributes to a constitutive “pro-HR” environment. HR levels were evaluated upon enzymatic induction of a DSB in a reporter construct, implicating that the bias between HR and NHEJ in resistant cells is not restricted to the context of ICL repair. However, it might be cell-cycle dependent. Importantly, we did not observe a reduction of 53BP1 foci following IR in the mother and sister cells compared to control (Figure 2C and 2D). With a majority of irradiated cells in G1, it might be difficult to visualize a G2-restricted decrease of 53BP1 foci.

In order to decipher the precise mechanism involved, we aimed to identify the genetic origin of the attenuation of MMC sensitivity. Among the results of WES analysis performed to this purpose, the R32C mutation of ATM arose as our best hit. Indeed, the key contribution of this kinase to DDR has been extensively described [22, 23]. ATM phosphorylates a multitude of targets to coordinate cell cycle checkpoints and DNA repair in response to DSBs. More precisely, it has been shown that ATM mediates the phosphorylation of 53BP1 at several S/T-Q sites, which is necessary for the recruitment of RIF1 at DSBs and subsequent repair by NHEJ [9, 10, 24]. On the other hand, the cell-cycle dependent phosphorylation of CtIP by ATM promotes DSB repair by HR [25]. Thus, ATM seems to play a central role in the choice between NHEJ and HR for the repair of DSBs.

Using CRISPR/Cas9 technology, we disrupted the R32C allele in ATM heterozygous cells from the mother. If the resistance to MMC were to be caused by this ATM mutation, one would expect a normalized MMC sensitivity in ATM –/WT cells. But both ATM R32C/WT and ATM –/WT presented the same increased survival when exposed to MMC. We concluded that the R32C allele was dispensable to induce the resistance phenotype. However, we cannot exclude the hypothesis that a decrease in ATM activity due to the R32C mutation or reduced levels of ATM due to allele loss would end up to the same phenotype in regard to ICL sensitivity. Recently, it has been shown that a competition between 53BP1 phosphorylation by ATM and dephosphorylation by PP4C defines the choice between NHEJ and HR in G2 [21]. According to these data, a reduced ability of ATM to phosphorylate 53BP1 in this specific context, either caused by less ATM or less activity of mutated ATM, could promote faster 53BP1 repositioning and favor HR over NHEJ.

We analyzed the functionality of R32C ATM using an ATM –/R32C human fibroblast cell line or R32C homozygous mice. Our data demonstrate that this variant retains its kinase activity, contributes to a normal DDR upon IR-induced damage, and does not impair the immune system development in mice. The R32C mutation could induce subtle modifications in one of the many functions of ATM that would require deeper analysis to be discovered.

To conclude, this work, in accordance with previous findings, further underlines the necessity for cells challenged with ICLs to favor HR over NHEJ. Our study also highlights the role of the DDR factor 53BP1 in the regulation of DSB repair pathway choice, since a reduced recruitment of this protein at ICLs can result in an increase resistance to MMC. Furthermore, we provide here evidence that ICL cellular toxicity can be significantly decreased without major alteration of the NHEJ pathway. This raises the possibility that fine-tuning the balance between DSB repair pathways could help to control the genomic instability of FA cells.

## MATERIALS AND METHODS

### Cell culture and transfection

Informed consent for our study was obtained from the family in accordance with the Helsinki Declaration. The Institut National de la Santé et de la Recherche Médicale Institutional Review Board also approved this study. Primary fibroblasts were obtained through culture of skin biopsies from all 5 members of the studied family, and further SV40-transformed and telomerase-immortalized as previously described [26]. Control fibroblasts were from healthy donors. Cernunnos-deficient fibroblasts were described previously [26]. Cells were grown in RPMI medium supplemented with 10% FBS and penicillin/streptomycin. Cell transfection was performed using the NEPA21 electroporator. For complementation studies, wt FANCG orf was cloned in front of an ires-EGFP cassette into the EGFP lentivirus vector pLenti7.3 (Life technologies). Viral supernatant production and cell transductions were performed as previously described [27].

### Western blot

Cells were lysed for 20 min on ice in lysis buffer (50mM Tris pH 8.0, 1% NP40, 2mM EDTA) supplemented with complete mini protease inhibitor cocktail Tablets (Roche) and phosphatase inhibitors cocktails 1 and 2 (Sigma). Whole cell lysates were analyzed by SDS-PAGE and blotted with the following antibodies: polyclonal rabbit anti-FANCD2 (Santa Cruz sc-28194); monoclonal mouse anti-ATM (clone 2C1, Santa Cruz sc-23921); monoclonal rabbit anti-phospho-ATM S1981 (Novus NB110-66655); polyclonal rabbit anti-phospho-Kap1 S824 (Bethyl Laboratories IHC-00073) and mouse monoclonal anti-Ku70 (Santa Cruz sc-17789).

### G2/M arrest analysis

Early passages primary fibroblasts were treated with increasing doses of MMC and harvested 48 hours later. The cells were then fixed, stained using propidium iodide, and analyzed by flow cytometry as previously described [27].

### Cell survival assays

SV40-transformed fibroblasts were plated on 24-well plates and either treated with MMC or phleomycin, or X-ray irradiated. Cells were collected 7 days after DNA damage induction, and the number of live cells was counted by flow cytometry. The percentage of survival was calculated relative to untreated cells.

### Immunofluorescence

Primary fibroblasts grown on glass coverslips were either X-ray irradiated (0.5Gy) or pulsed with 1 μg/ml MMC for 1 hour. After PBS wash and medium renewal, the cells were incubated for the indicated times before fixation and staining as previously described [26]. The following primary antibodies were used: polyclonal rabbit anti-53BP1 (clone H-300, Santa Cruz sc-22760); anti-γH2AX (Millipore 05-636); monoclonal mouse anti-BRCA1 (Santa Cruz sc-6954) and monoclonal rabbit anti-phospho-ATM S1981 (Novus NB110-66655). The AlexaFluor 488 secondary antibody and DAPI were from Molecular Probes. Slides were viewed on a ZEISS Axioplan epifluorescent microscope using a 63x objective. Image processing and foci quantification were performed on ImageJ software (http://rsb.info.nih.gov/ij/).

### HR assay

SV40-transformed and telomerase-immortalized fibroblasts were transfected with the pHPRT-DRGFP plasmid (Addgene plasmid 26476; gift from Maria Jasin) [28] and grown under puromycin selection to obtain DR-GFP cell lines with a stably integrated copy of the reporter. DR-GFP cell lines seeded in a 12-well plate were then co-transfected with 0.75 μg I-SceI and 0.075 μg RFP expressing plasmids using jetPRIME reagent (Polyplus). The 10:1 ratio assured that all the RFP^+^ cells were transfected with the I-SceI plasmid. Upon expression of I-SceI in the transfected cells, HR between the non-functional *iGFP* and *SceGFP* genes of the DRGFP substrate allows expression of a functional GFP gene. Cells were harvested 72 hours post transfection and analyzed by flow cytometry. The percentage of HR was determined by the percentage of GFP^+^ cells among the transfected cells (i.e. RFP^+^ cells) to normalize the results according to the transfection efficiency.

### Whole-exome sequencing

Whole-exome sequencing was performed through 100-bp paired-end reads on Illumina HiSeq after capture using an exome enrichment kit (TruSeq; Illumina) as previously described [29].

### *In vitro* CRISPR/Cas9 genome engineering

The 5′-AAACAATTAAACATCTAGAT-3′ sequence located in human ATM exon 3 was used as gRNA and cloned into the pX330-U6-Chimeric_BB-CBh- hSpCas9 plasmid (a gift from F. Zhang, the Broad Institute of Massachusetts Institute of Technology and Harvard University, Cambridge, MA; Addgene plasmid 42230) [30] for transfection into SV40-transformed and telomerase-immortalized fibroblasts of the mother. Clones obtained after limiting dilution of the bulk of transfected cells were screened by sequencing.

### G2/M checkpoint analysis

G2/M checkpoint analyses were performed as previously described [26]. The rabbit polyclonal anti-phospho-H3 antibody was from Millipore (06-570).

### Generation of the ATM R32C mouse line

All transgenesis experiments were carried out by the Centre d’Ingénierie Génétique Murine of the Pasteur Institute and performed in accordance with the European Community guidelines (2010/63/UE) and with French national regulations for the care and use of laboratory animals.

The ATM R32C mouse line was obtained on a C57BL/6xDBA2 genetic background by co-microinjection of 5ng/μL of circular pX330-based CRISPR/Cas9 plasmid and 100 ng/μL of a single-stranded oligonucleotide DNA donor matrix into one-cell stage embryos.

The plasmid was obtained by cloning the 5′-TAAATTTAAGCGCCTGATTC-3′ gRNA located in exon 3 of murine ATM into the pX330-U6-Chimeric_BB-CBh- hSpCas9 plasmid (a gift from F. Zhang, the Broad Institute of Massachusetts Institute of Technology and Harvard University, Cambridge, MA; Addgene plasmid 42230) [30]. The repair template was a 134-nt single-stranded oligonucleotide homologous to the targeted sequence expect for 5 mismatches that would introduce the c.94C>T mutation to create the R32C allele, a G>A silent mutation to disrupt the PAM sequence (c.105G>A) and 3 silent mutations allowing the screening of the recombinant animals (c.93G>A, c.99G>C, c.102T>C) (Supplementary Figure 2A).

Microinjected embryos were implanted into the oviducts of C57BL/6J×CBA F1 foster mothers following standard procedures [31]. Out of 67 new-borns, 47 (70%) had at least one mutated allele, among which 6 (9%) had an allele correctly recombined with the 5 mutations introduced by the oligonucleotide. Three of these recombinant mice were bred to C57B6/N WT mice to establish the ATM R32C heterozygous mouse line and its progeny was intercrossed to obtain ATM R32C homozygous mice. One F0 mice with a KO ATM allele was also bred to C57B6/N WT mice and the progeny was intercrossed to generate an ATM KO mouse line.

All experiments and procedures were submitted for approval to the French Ministry of Agriculture’s Regulation for Animal Experimentation (act 87847, 19 October 1987; modified in May 2001).

### Mice immunophenotyping

Immunophenotyping was performed on thymic and splenic lymphoid populations by five-color fluorescence analysis, according to standard protocols. The following anti-mouse antibodies were used: CD8-PE (catalog number 553041), IgM-APC (catalog number 550676), CD4-PE.Cy7 (catalog number 552775), CD25 PerCPCy5.5 (catalog number 551071) from BD Biosciences, Franklin Lakes, NJ, USA; B220-PercPCy5.5 (catalog number 1116180) and CD44 BV510 (catalog number 1115215) from Sony Biotechnology Inc., San Jose, CA, USA. Cells were analyzed on a LSR FORTESSA X-20 cytometer (BD Biosciences) immediately after incubation with Molecular Probes Sytox Blue Dead Cell Stain (catalog number S34857) (Life Technologies) to exclude dead cells.

### Irradiation of activated mouse T-cells

Splenocytes were isolated and activated with GibcoDynabeads Mouse T-activator CD3/CD28 beads (catalog number 11452D) (Life Technologies) according to the manufacturer’s recommendations, for 3 days. The activated T-blasts were irradiated at 10 Gy before lysis in RIPA buffer (50 mM Tris pH 7,5, 150 nM NaCl, 1% Triton X100, 1 mM EDTA, 0,25% Sodium Deoxycholate) and analysis of the whole cell extracts by Western Blot.

## SUPPLEMENTARY MATERIALS FIGURES


