# Supplementary material for: Reduced recruitment of 53BP1 during interstrand crosslink repair is associated with genetically inherited attenuation of mitomycin C sensitivity in a family with Fanconi anemia
Source: Oncotarget. 2017 Dec 17;9(3):3779–93. doi: 10.18632/oncotarget.23375 (PMC5790499; doi:10.18632/oncotarget.23375)
Supplement: Supplementary file 1 [file oncotarget-09-3779-s001.pdf]

Reduced recruitment of 53BP1 during interstrand crosslink repair is associated with genetically inherited attenuation of mitomycin C sensitivity in a family with Fanconi anemia

SUPPLEMENTARY MATERIALS

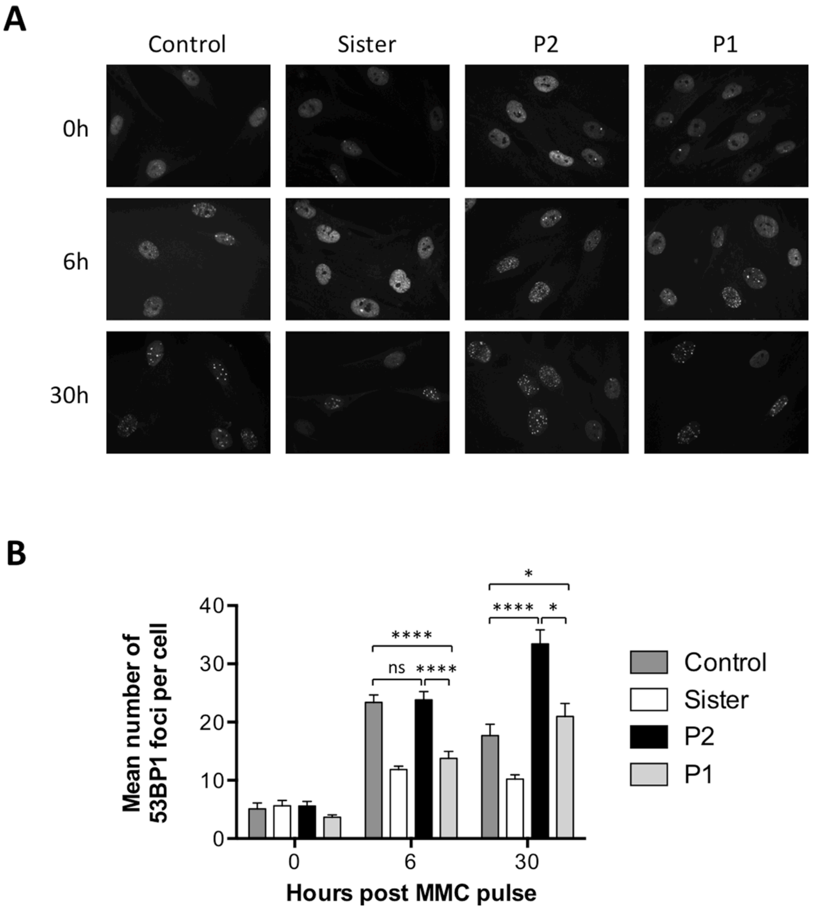

Supplementary Figure 1: The recruitment of 53BP1 at ICLs is delayed in P1 compared to P2. (**A–B**) Recruitment of 53BP1 following MMC treatment. Primary fibroblasts from patients P1, P2, their sister, and a healthy control were pulsed for 1 hour with 1 µg/ml MMC and further incubated for the indicated times before fixation and 53BP1 immunostaining. (A) Representative images. (B) Number of foci per cell as quantified from at least 100 nuclei from 3 independent experiments, shown as mean ± SEM. Statistical analyses were performed using Mann-Whitney tests. $^{*}P < 0.01$; $^{****}P < 0.0001$; ns: not significant.

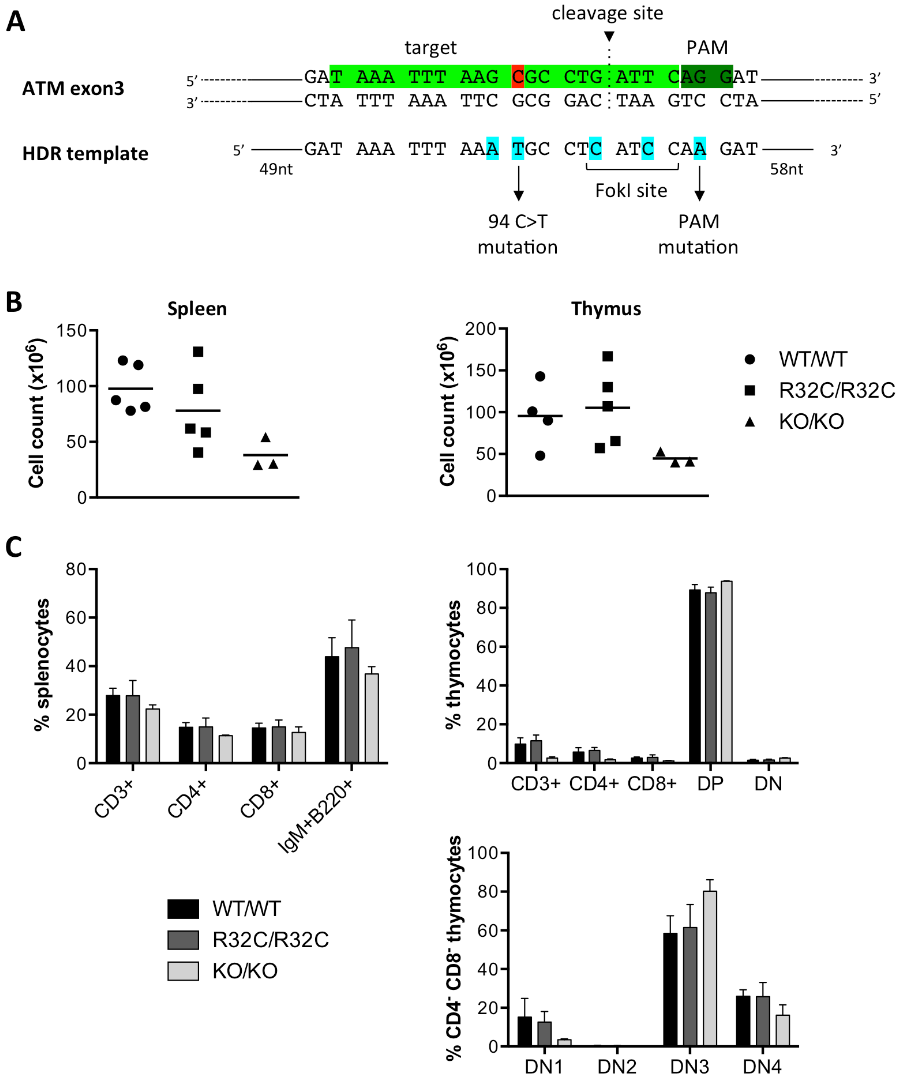

Supplementary Figure 2: Generation and immunophenotyping of ATM R32C homozygous mice. (**A**) Sequences of the CRISPR-Cas9 target in exon 3 of mouse ATM and of the HDR template used to generate the ATM R32C mouse line. (**B–C**) Immunophenotyping results obtained from ATM WT/WT (*n* = 5), R32C/R32C (*n* = 5) or ATM KO/KO (*n* = 3) mice. (B) Splenocytes and thymocytes cell counts. (C) Percentages of immune cell populations determine by flow cytometry after CD3/CD4/CD8/B220/IgM or CD3/CD4/CD8/CD44/CD25 staining of splenocytes or thymocytes respectively. DP: double positive (CD4+ CD8+). DN: double negative (CD4– CD8–). Percentages of DN1 (CD44+CD25–), DN2 (CD44+CD25+), DN3 (CD44–CD25+) and DN4 (CD44–CD25–) cells were calculated among the DN thymocytes.

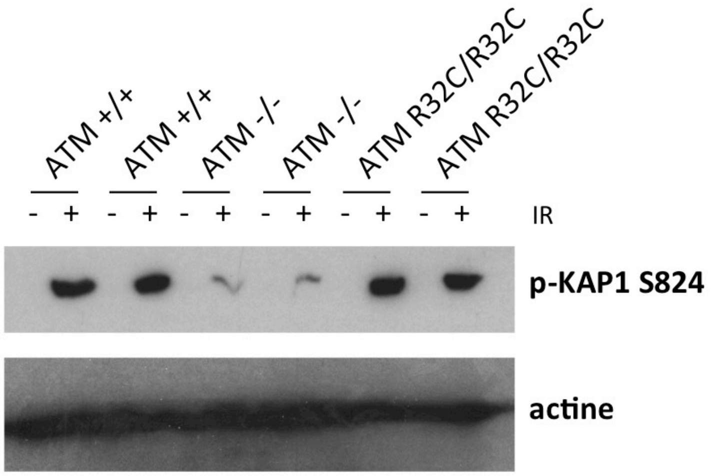

Supplementary Figure 3: T cells from ATM R32C homozygous mice have a normal response to IR. CD3/CD28 activated splenocytes from ATM +/+, –/– or R32C/R32C mice were irradiated and cell extracts were analyzed by phospho-Kap1 S824 immunoblot. Actine immunoblot served as loading control.
